# An Extension Network of Dendritic Neurons

**DOI:** 10.1155/2023/7037124

**Published:** 2023-01-23

**Authors:** Qianyi Peng, Shangce Gao, Yirui Wang, Junyan Yi, Gang Yang, Yuki Todo

**Affiliations:** ^1^Faculty of Engineering, University of Toyama, Toyama-shi 930-8555, Japan; ^2^Faculty of Electrical Engineering and Computer Science, Ningbo University, Ningbo, Zhejiang 315211, China; ^3^Department of Computer Science & Technology, Beijing University of Civil Engineering and Architecture, Beijing 100044, China; ^4^School of Information, Renmin University of China, Beijing, China; ^5^Faculty of Electrical, Information and Communication Engineering, Kanazawa University, Kanazawa, Ishikawa 9201192, Japan

## Abstract

Deep learning (DL) has achieved breakthrough successes in various tasks, owing to its layer-by-layer information processing and sufficient model complexity. However, DL suffers from the issues of both redundant model complexity and low interpretability, which are mainly because of its oversimplified basic McCulloch–Pitts neuron unit. A widely recognized biologically plausible dendritic neuron model (DNM) has demonstrated its effectiveness in alleviating the aforementioned issues, but it can only solve binary classification tasks, which significantly limits its applicability. In this study, a novel extended network based on the dendritic structure is innovatively proposed, thereby enabling it to solve multiple-class classification problems. Also, for the first time, an efficient error-back-propagation learning algorithm is derived. In the extensive experimental results, the effectiveness and superiority of the proposed method in comparison with other nine state-of-the-art classifiers on ten datasets are demonstrated, including a real-world quality of web service application. The experimental results suggest that the proposed learning algorithm is competent and reliable in terms of classification performance and stability and has a notable advantage in small-scale disequilibrium data. Additionally, aspects of network structure constrained by scale are examined.

## 1. Introduction

In recent years, deep learning (DL) has dominated the research field of artificial intelligence (AI) and achieved dramatic successes in terms of speech recognition, protein structure prediction, drug discovery, image and video processing, and others. [1]. At present, the mainstream deep learning models are mostly constructed based upon neural networks, referring to multiple-layered parameterized McCulloch–Pitts neurons inspired by the biological neuron [2]. Neural networks as black boxes are not only extensively studied in the field of artificial intelligence but also highly applied to the industry of information technology [3, 4]. The appearance of deep neural networks pushes the development of neural networks to a new peak. However, given the numerous difficulties and problems they face, including the lack of a theoretical foundation for explanation [5, 6], fairness [7, 8], and causal discovery [9, 10], neural networks tend to become stuck in a relatively inert state. In pace with the increasing attention of interpretative theory, it is essential to urgently require newer and better discoveries and more valuable research orientations to avoid blindness for the promotion of scientific and technological progress.

From the perspective of understanding the mechanics of artificial neural networks, various methods and contributions are introduced [11, 12]. Through the application of the Monte Carlo simulation for quantifying the variable's importance, Olden et al. justified the rightness of the connection weight calculation method in neural networks [13]. Statistics pointed out that Sarle et al. proclaimed the relations between neural networks and the generalized linear model, maximum redundancy analysis, projection tracking, clustering analysis, and other statistical models and also transformed terms in neural networks into statistical terms [14]. Similarly, certain relations between artificial neural networks and statistical methods were proposed in [15].

**Figure 1:**
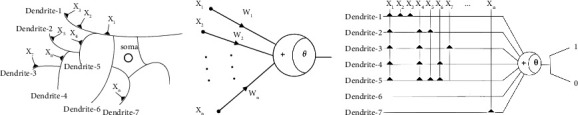
(a) The function of a biological neuron is completely different depending on the shape of its dendrites and the location of its synapses. (b) McCulloch–Pitts neuron model: no interaction in dendrite morphology and dendrites. (c) Single dendritic neural model: faithful representation of dendrite morphology and dendrites fixated on binary classification.

Despite putting forward the abovementioned studies, the bottleneck of neural networks is not addressed. Originating from the simulation of neurons in biological concepts, the artificial neural network takes artificial neurons as nodes to construct a complete conduction structure. As a basic information processing unit, the neuron is formed by a dendrite, cell body (soma), axon, and synapse, as shown in Figure 1(a). To be specific, the dendrite receives information from the outside world, the cell body processes the information, and the axon and synapse transmit the signal and pass it on to other neurons, respectively [16]. The structure of biological neurons can be traced back to the 1940s, when McCulloch and Pitts jointly published the abstract neuron model McCulloch–Pitts (MP) [17] for the first time, as illustrated in Figure 1(b). Then, in 1949, the Hebb rule [18] was proposed based on the theory of the variability of synapse connections of neurons within the human brain. The adjusting weight method was introduced into machine learning, thereby laying the foundation for the learning algorithms.

Inspired by the structure of biological neurons and MP, Todo et al. proposed the dendritic neural model (DNM) [19]. Different from the traditional neural network model, the dendritic neural model is designed based on neuron conduction and single neuron. DNM obtains the support of the biological theory to simulate the biological neuron. Furthermore, DNM compensates for some defects of the homologous perceptron model, such as the inability to solve the XOR problem. At the same time, the novel study of the human brain [20] is also brought about.

As a classifier, shown in Figure 1(c), DNM has been applied to various classification problems. For example, Sha et al. classified the breast cancer dataset, and Jiang et al. detected the liver disorder [21] for assisted disease diagnosis. Apart from the development of medical aid applications, an unconventional method was also applied to the financial field. To improve the classification performance, metaheuristic algorithms were introduced to train the hyperparameters of DNM [22, 23]. Through the use of the decision tree, Luo et al. initialized the model to realize better effectiveness [24]. For solving a generalized large-scale classification problem, Jia et al. suggested a reconciliation method with DNM by using a particle antagonism mechanism, and Ji et al. proposed a DNM-based multiobjective evolutionary algorithm [25]. In terms of feature selection, Song et al. addressed the high-dimensional challenge [26], and Gao et al. also showed the expansibility and flexibility of DNM for diverse applications [27]. Utilizing the multiplication operation that is useful to the information processing for a single neuron, the computing in synapses is imaginatively described using sigmoid functions. It is advantageous to establish the morphology of a neuron by determining the values of the parameters in synapses since the output of synapses can effectively represent signals. Nevertheless, it is noted that the single neuron is limited in partial application scenarios. In [28], the binary classification results of DNM were incorporated to undertake multiple classification tasks and thus recognize the multiclassification datasets.

**Figure 2:**
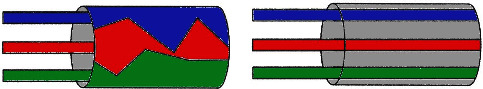
(a) The network is disordered without QoS rules. (b) The network is in order with QoS rules.

By adopting the quality of service (QoS) as the evaluation dataset, this study implements the multiclassification of web service selection. QoS is defined as the fact that a network utilizes a range of basic technologies to provide superior service capabilities for the designated network communication [29]. As a security mechanism of the network and a technology, QoS is carried out to deal with network delay [30], blocking [31], and other problems. For a general situation, the common network bandwidth as a significant metric is instanced in order to illustrate QoS. When the standard of service quality has not appeared, the network environment treats all services and applications in an equal way, resulting in a disordered situation, as shown in Figure 2(a) where the colored area stands for different web services and applications. In other words, when a network device does not have the capacity of QoS, the network environment will be threatened, and a bottleneck will be created [32]. As shown in Figure 2(b), prioritization from the perspective of QoS provides a more orderly, efficient, and stable network environment. QoS contains a set of nonfunctional attributes, which is the measure and criteria of such characteristics of the web services, such as reliability and response time, to effectively classify and sort different services.

Web services refer to some software modules running on the network, which are service-oriented and based on distributed programs. Due to the fact that the web service employs general Internet standards, such as HTTP and XML (a subset of the standard generalized markup language) [33], human beings then have access to data on the web via various terminal devices in various places. In this article, the described web service is different from the common network application. It generally refers to some application modules, such as the network protocol and method, which is the basis of network applications. With the development of the Internet, many candidate services have implemented the same task, and most of them have the same functions but different nonfunctional characteristics. As a result, these services are divided into different service quality levels. Overlapping is seen to be inevitable because of the existence of a wide range of web services on the network. Based on the QoS, web service selection is considered an effective solution [34]. As network technology and operation concepts develop rapidly, web services are becoming the latest technology and development trend for constructing distributed, modular, and service-oriented applications.

**Figure 3:**
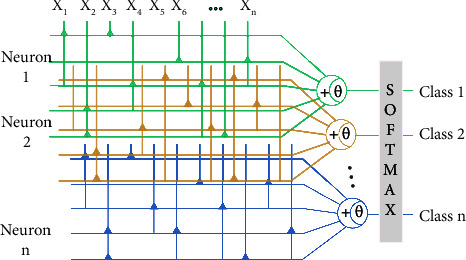
Multiple dendritic neural networks: applied on comprehensive applications based on the dendritic structure.

Based on DNM, this article proposes a multiple dendritic neural network (MDNN) with multiple single neurons to achieve the multiclassification of web service selection based on QoS. To adjust the multiclassification mechanism, the structure of DNM introduced in Figure 3 is reconstructed. For the purpose of accelerating the gradient descent and improving the multiclassification accuracy, the backpropagating algorithm and adaptive moment estimation optimization are derived for the first time. Experiments are carried out on the Quality of Web Service dataset and nine UCI multiclassification datasets [35]. In the comparison between MDNN and nine state-of-the-art classifiers, the superiority of the proposed method is demonstrated.

The contributions are majorly classified into the aspects as follows: 1) a novel multiple single-neuron neural network for multiclassification tasks is developed. 2) The potential and application scenarios of the dendritic neural network are explored. 3) A new approach for QoS-driven web service selection is proposed.

Given as follows is the organization of the remaining parts of this article: Section 2 presents the structure of the multiple dendritic neural networks.  Section 3 elaborates on the learning processes of the proposed method and expounds on the optimization strategies. The comparison with other algorithms and experimental results are shown in Section 4. At last, Section 5 concludes the paper and formulates future work.

## 2. The Dendritic Neuron Network-Based Multiclassifier Approach

**Figure 4:**
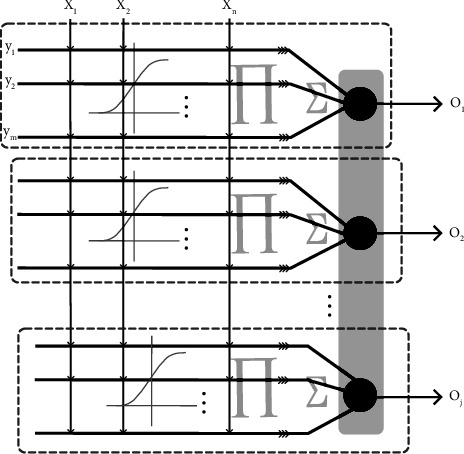
The structure of the proposed MDNN. The framed rectangle represents the structure of a single neuron.

The proposed multiclassifier is constructed by multiple single neurons. The general architecture is shown in Figure 4. As for each neuron, *x*_*i*_, the input of the model is preprocessed by using a nonlinear sigmoid filter. To differentiate neurons, the function introduces the subscript *j*, which is defined as follows:

(1) $$\begin{array}{c} Y_{j,i,m}=\frac{1}{1+e^{-\left(w_{j,i,m}x_{i}-q_{j,i,m}\right)}}\text{,} \end{array}$$

where *i* is the number of attributes of the sample, *m* is the number of nodes within the hidden layer, and *j* is the number of classifications of output results. In addition, the weight *w*_*j*,*i*,*m*_ and threshold *q*_*j*,*i*,*m*_ denote the neural network parameters in the training stage and are randomly initialized within (0, 0.01) and 0, respectively.

In contrast to the perceptron model, a quadrature method is adopted for the hidden layer to not only rule out the inhibited neuronal excitation but also enhance the activated neuronal excitation. The formula is described as follows:

(2) $$\begin{array}{c} Z_{j,m}=\prod_{i=1}Y_{j,i,m}\text{,} \end{array}$$

(3) $$\begin{array}{c} V_{j}=\underset{m=1}{\sum }Z_{j,m}\text{.} \end{array}$$

Eq. (2) means that all of the hidden layers are activated, which is equivalent to a logical AND. Eq. (3) is equivalent to a logical OR where all inhibited neuronal excitations from the former layer are suppressed exclusively and the rest are reactivated. As a result, the multiclassification structure of multiple neurons is formed.

Apart from the dendritic mechanism, MDNN utilizes the normalized exponential function to output final results. For ease of consistency in representation, the illustrated style of the normalized exponential function is followed. To be noted, the output of multiple neurons is processed by all information from the previous layer instead of being directly conveyed, expressed as follows:

(4) $$\begin{array}{c} O_{j}=\frac{e^{V_{j}}}{\sum_{j=1}e^{V_{j}}}\text{,} \end{array}$$

where *O*_*j*_ is the possibility of the prediction for each class. The normalized exponential function converts the output value of the upper layer, *V*_*j*_, to the probability distribution with the range of [0, 1], and the sum of the probability values of each neuron being 1. The formula first converts the results *V*_*j*_ into an exponential function, ensuring the nonnegative probability, and then normalizes the probability values into 1.

Since the prediction result follows the rules of the probability distribution, the cross-entropy function as the loss function is considered a proper substitute for mean square error, which is defined as follows:

(5) $$\begin{array}{c} E_{j}=-\underset{j=1}{\sum }T_{j}*\;\log O_{j}\text{,} \end{array}$$

where *E*_*j*_ represents the similarity of probability distribution between the prediction of the model and the actual classification and *T*_*j*_ is the actual classification label.

## 3. Learning Mechanism and Optimization Strategies

The existing learning algorithms cannot be directly applied since MDNN is a new dendritic neuron model containing multiplication operators in its calculation. Accordingly, in this section, we for the first time derive the learning algorithms for our proposed MDNN, specifically one is the traditional error backpropagation, and the other is an Adam-like learning algorithm.

### 3.1. Backpropagation

In the course of learning samples, the model is promoted by the stochastic gradient descent of parameters *w*_*j*,*i*,*m*_ and *q*_*j*,*i*,*m*_, which is described as follows:

(6) $$\begin{array}{c} w_{j,i,m}^{t}=w_{j,i,m}^{t-1}-\eta {∆w}_{j,i,m,}\\ q_{j,i,m}^{t}=q_{j,i,m}^{t-1}-\eta ∆q_{j,i,m}\text{,} \end{array}$$

where *η* as the learning rate is a positive constant. *t* and *t* − 1 denote the current iteration and the previous iteration in the training stage, respectively.

**Figure 5:**
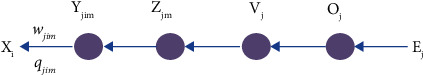
The relation among layers.

The error of the proposed MDNN is calculated by the cross-entropy function. According to the calculated error, the error backpropagation algorithm is introduced as the learning scheme. In backpropagation, all the samples or a batch of samples are involved. To better realize intuition, the relation among layers is shown in Figure 5.

∆*w*_*j*,*i*,*m*_ and ∆*q*_*j*,*i*,*m*_ are expressed by the partial differential form as follows:

(7) $$\begin{array}{c} {∆w}_{j,i,m}=\frac{\partial E_{j}}{\partial O_{j}}\frac{\partial O_{j}}{\partial V_{j}}\frac{\partial V_{j}}{\partial Z_{j,m}}\frac{\partial Z_{j,m}}{\partial Y_{j,i,m}}\frac{\partial Y_{j,i,m}}{\partial w_{j,i,m}}\text{,}\\ ∆q_{j,i,m}=\frac{\partial E_{j}}{\partial O_{j}}\frac{\partial O_{j}}{\partial V_{j}}\frac{\partial V_{j}}{\partial Z_{j,m}}\frac{\partial Z_{j,m}}{\partial Y_{j,i,m}}\frac{\partial Y_{j,i,m}}{\partial q_{j,i,m}}\text{.} \end{array}$$

Since the model is trained by batches, ∆*w*_*j*,*i*,*m*_ and ∆*q*_*j*,*i*,*m*_ obtained by the gradient descent are finally calculated as follows:

(8) $$\begin{array}{c} \bar{∆w_{j,i,m}}=\frac{∆w_{j,i,m}}{N}\text{,}\\ \bar{∆q_{j,i,m}}=\frac{∆q_{j,i,m}}{N}\text{,} \end{array}$$

where *N* denotes the size of input data within the current iteration.

Following the chain rule, the derivation procedures and results are presented according to the backpropagation. Firstly, the partial differential of error *E* is calculated. By the empirical evidence of normalized exponential function, *∂E*_*j*_/*∂O*_*j*_ and *∂O*_*j*_/*∂V*_*j*_ are computed collectively instead of computing them separately.

The forward propagation for the multiclassification is not directly corresponding. Thus, the derivation of error *E* is discussed in Cases (1) and (2). To avoid confusion, the subscripts of *V* and *O* are redefined as *m* and *n*, respectively. Their relation is simplified as follows:

(9) $$\begin{array}{c} V_{m}⟵O_{n}⟵E_{n}. \end{array}$$

On the basis of Equations. (5) and (4), when *n*=*m*, there is Case (1):

(10) $$\begin{array}{c} \frac{\partial E_{n}}{\partial V_{m}}=\frac{\partial E_{n}}{\partial O_{n}}\frac{\partial O_{n}}{\partial V_{m}}\text{,}\\ =-T_{n}\frac{1}{O_{n}}\left[O_{n}\left(1-O_{n}\right)\right]\text{,}\\ =-T_{n}\left(1-O_{n}\right)\text{.} \end{array}$$

When *n* ≠ *m*, there is Case (2):

(11) $$\begin{array}{c} \frac{\partial E_{n}}{\partial V_{m}}=-\underset{n\neq m}{\sum }T_{m}\frac{1}{O_{m}}\left(-O_{n}O_{m}\right)\\ =\underset{n\neq m}{\sum }T_{m}O_{n}\text{.} \end{array}$$

We incorporate Cases (1) and (2) into the following formula:

(12) $$\begin{array}{c} \frac{\partial E_{n}}{\partial V_{m}}=-T_{n}\left(1-O_{n}\right)+\underset{n\neq m}{\sum }T_{m}O_{n,}\\ =-T_{n}+T_{n}O_{n}+\underset{n\neq m}{\sum }T_{m}O_{n}\text{,}\\ =O_{n}\left(T_{n}+\underset{n\neq m}{\sum }T_{m}\right)-T_{n}\text{,}\\ =O_{n}-T_{n}\text{.} \end{array}$$

Thus, *∂E*_*j*_/*∂V*_*j*_ is expressed as follows:

(13) $$\begin{array}{c} \frac{\partial E_{j}}{\partial V_{j}}=\frac{\partial E_{j}}{\partial O_{j}}\frac{\partial O_{j}}{\partial V_{j}}=O_{j}-T_{j}\text{.} \end{array}$$

For the rest layers of MDNN, they are derived according to Equations. (3) and (2) as follows:

(14) $$\begin{array}{c} \frac{\partial V_{j}}{\partial Z_{j,m}}=1\text{,} \end{array}$$

(15) $$\begin{array}{c} \frac{\partial Z_{j,m}}{\partial Y_{j,i,m}}=\frac{Z_{j,m}}{Y_{j,i,m}}\text{.} \end{array}$$

Taking the derivative of Equation. (1) with the sigmoid function, *∂Y*_*j*,*i*,*m*_/*∂w*_*j*,*i*,*m*_ and *∂Y*_*j*,*i*,*m*_/*∂q*_*j*,*i*,*m*_ are obtained as follows:

(16) $$\begin{array}{c} \frac{\partial Y_{j,i,m}}{\partial w_{j,i,m}}=x_{i}\cdot Y_{j,i,m}\left(1-Y_{j,i,m}\right)\text{,}\\ \frac{\partial Y_{j,i,m}}{\partial q_{j,i,m}}=-Y_{j,i,m}\left(1-Y_{j,i,m}\right)\text{.} \end{array}$$

### 3.2. Adam-Like Optimization

For improving the convergence and classification ability of the proposed model, inspired by the well-known adaptive moment estimation (Adam) [36], an Adam-like learning algorithm for MDNN is also introduced to accelerate the gradient descent without diverging. The way of updating weights in each iteration is optional. The traditional way mentioned in Section 3.1 or Adam can be altered according to the user's setting.

As an extended optimization strategy of stochastic gradient descent (SGD) [37], momentum [38, 39] is introduced to reduce the oscillation and accelerate the gradient descent. The fundamental concept of gradient descent with momentum lies in updating the weight by calculating the exponentially weighted average of the gradient as follows:

(17) $$\begin{array}{c} v_{∆w_{j,i,m}}=\alpha v_{∆w_{j,i,m}}+\left(1-\alpha \right)∆w_{j,i,m}\text{,} \end{array}$$

(18) $$\begin{array}{c} v_{∆q_{j,i,m}}=\alpha v_{∆q_{j,i,m}}+\left(1-\alpha \right)∆q_{j,i,m}, \end{array}$$

where *α* is a positive constant to smooth out the gradient descent process. Intuitively, ∆*w*_*j*,*i*,*m*_ and ∆*q*_*j*,*i*,*m*_ are interpreted as the acceleration in physics. *v*_∆*w*_*j*,*i*,*m*__ and *v*_∆*q*_*j*,*i*,*m*__ are regarded as the velocity, and *α* is seen as the friction. In addition, ∆*w*_*j*,*i*,*m*_ and ∆*q*_*j*,*i*,*m*_ accelerate the gradient descent and gain the velocity ∆*w*_*j*,*i*,*m*_ and ∆*q*_*j*,*i*,*m*_, and the friction *α* prevents the acceleration.

In this case, the updates of parameters *w*_*j*,*i*,*m*_ and *q*_*j*,*i*,*m*_ are modified as follows:

(19) $$\begin{array}{c} w_{j,i,m}^{t}=w_{j,i,m}^{t-1}-\eta v_{∆w_{j,i,m}}\text{,} \end{array}$$

(20) $$\begin{array}{c} q_{j,i,m}^{t}=q_{j,i,m}^{t-1}-\eta v_{∆q_{j,i,m}}\text{.} \end{array}$$

Serving as a crucial part of Adam, the root mean square prop (RMSprop) [40] auxiliary accelerates the gradient descent as follows:

(21) $$\begin{array}{c} u_{∆w_{j,i,m}}=\beta v_{∆w_{j,i,m}}+\left(1-\beta \right)∆w_{j,i,m}^{2}\text{,} \end{array}$$

where *β* is a positive constant similar to *α*. *w*_*j*,*i*,*m*_ is calculated as follows:

(22) $$\begin{array}{c} w_{j,i,m}^{t}=w_{j,i,m}^{t-1}-\eta \frac{∆w_{j,i,m}}{\sqrt{v_{∆w_{j,i,m}}}}\text{.} \end{array}$$

Similarly, *u*_∆*q*_*j*,*i*,*m*__ is obtained by

(23) $$\begin{array}{c} u_{∆q_{j,i,m}}=\beta v_{∆q_{j,i,m}}+\left(1-\beta \right)∆q_{j,i,m}^{2}\text{,} \end{array}$$

(24) $$\begin{array}{c} q_{j,i,m}^{t}=q_{j,i,m}^{t-1}-\eta \frac{∆q_{j,i,m}}{\sqrt{v_{∆q_{j,i,m}}}}\text{.} \end{array}$$

In order to avoid the bias of exponentially weighted average in the initial learning stage, Equations. (17), (18), (21), and (23) are modified to obtain more accurate results as follows:

(25) $$\begin{array}{c} v_{corr}^{∆w_{j,i,m}}=\frac{v_{∆w_{j,i,m}}}{1-\alpha^{t}}\text{,}\\ v_{corr}^{∆q_{j,i,m}}=\frac{v_{∆q_{j,i,m}}}{1-\alpha^{t}}\text{,}\\ u_{corr}^{∆w_{j,i,m}}=\frac{u_{∆w_{j,i,m}}}{1-\beta^{t}}\text{,}\\ u_{corr}^{∆q_{j,i,m}}=\frac{u_{∆q_{j,i,m}}}{1-\beta^{t}}\text{.} \end{array}$$

For the acceleration of the gradient descent, Adam combines RMSprop with momentum. Thus, based on Equations. (23) and (24), the parameter updating equations optimized by Adam are expressed as follows:

(26) $$\begin{array}{c} w_{j,i,m}^{t}=w_{j,i,m}^{t-1}-\eta \left(\frac{v_{corr}^{∆w_{j,i,m}}}{\sqrt{u_{corr}^{∆w_{j,i,m}}}+\varepsilon }\right)\text{,}\\ q_{j,i,m}^{t}=q_{j,i,m}^{t-1}-\eta \left(\frac{v_{corr}^{∆q_{j,i,m}}}{\sqrt{u_{corr}^{∆q_{j,i,m}}}+\varepsilon }\right)\text{,} \end{array}$$

where *ε* is an infinitesimal so as to prevent the computation overflow.

## 4. Experimental Evaluation

### 4.1. Experimental Setup

The quality of web service (QWS) dataset [41, 42] is a real-world dataset based on the quality of service. Several versions of QWS are available. In this study, experiments use the original version, which consists of 364 web services. Its quality is described by a total of 10 nonfunctional attribute indexes. The QWS dataset divides the web service into 4 levels from the highest to the lowest, which are platinum, gold, silver, and bronze.

To avoid overfitting while improving the accuracy of results, data preprocessing strategies are adopted in the experiments. The raw data are normalized by using the rule of standardization:

(27) $$\begin{array}{c} x=\frac{x-\bar{x}}{\sigma }\text{.} \end{array}$$

Moreover, the normalized data are randomly divided into three parts: 70 percent for the training process, 15 percent for the testing process, and the remaining data for the validation, to reduce unnecessary time consumption.

### 4.2. Optimal Parameter Settings

All of the hyperparameters are illustrated in Table 1, which presents their description and values. Sigmoid, tanh, Rectified Linear Unit (ReLU), and Leaky ReLU are available to freely choose the suitable active function. For large datasets, the samples are divided into mini-batch and shuffled for the gradient descent during the training stage. If batchsize is equal to the number of samples during the iteration, then the batch gradient descent will be executed.

However, it is tricky to determine the epoch size. To enable the model to reach optimal performance, a self-adaptive appending training epoch is arranged in the training stage according to the convergence of the validation process. Beginning with the default configuration, the epoch then adaptively increases pivoting on whether the gradient descent is approaching stagnation. At the same time, the initial value is set to 100 to avoid a higher epoch causing the time consumption. For general neural networks, experimental results are highly affected by the combination of parameters. Therefore, parameters, which are batchsize, *M*, *η*, and precision, are adjusted by the orthogonal experiment with 4 factors and 3 levels. The specific design is listed in Table 2. The optimal parameters of MDNN for QWS are finally shown in Table 3. The other parameters comply with the setting in Table 1.

### 4.3. Performance and Discussion

This section is divided into two subsections: experimental results of QWS analyzed by a variety of evaluation indicators are elaborated in Section 4.3.1, and Section 4.3.2 compares the proposed model with other multiclassification methods to demonstrate the prominent superiority of the proposed model.

#### 4.3.1. Experimental Results

For the comprehensive performance evaluation of MDNN, the following statistical indicators are used: precision, recall, *F*_1_ score, accuracy, and area under the curve (AUC) [43]. It is worth noting that the precision refers to the classification precision. For not only the convenience of the intuitive evaluation but also the justification of the following comparison, the macroaverage, which is the arithmetic average of performance indicators of all categories instead of instances, is adopted to statistically process classification results.

Table 4 shows the classification results of MDNN on the 4-class QWS dataset. The average values and optimum values represent the classification performance of MDNN. Although the dataset is technically unbalanced, such as the number of classes of QWS in Table 5, the stability and generalization ability of MDNN are considered to be effectively validated.

The five statistical indicators suggest that the classification achieved by MDNN for each class is effective, stable, and reliable. In their mean values, it is indicated that MDNN has good classification performance. The gradient descent optimization strategy effectively reduces the errors. Moreover, MDNN accelerates the gradient descent to maintain a continuous downward trend, thereby finally guaranteeing the generalization and robustness of MDNN.

#### 4.3.2. Comparison of Methods

To further verify the efficiency of MDNN, nine classifiers in total are used to compare with MDNN on nine multiclassification datasets from the UCI machine learning repository and the QWS dataset. The information of datasets and parameter settings of MDNN are listed in Table 5. The nine classifiers consist of BP, SVM, KNN, CART, naïve Bayes, LDA, QDA, J48, and random forest [44]. In addition, the ten datasets include Iris, Wine, Vehicle, Balance scale, CMC, Seed, Vowel, Thyroid, Robot navigation, and QWS.

Experimental results are shown in Table 6 where the best result for each dataset among all compared methods is highlighted in bold. According to five statistical indicators, it can be found that MDNN has the most optimal values in comparison with other classifiers. On the Iris, Wine, Seed, and Thyroid datasets, MDNN gives the best performance, with perfect outcomes of 100 percent correctness. Also, MDNN performs well with unbalanced data such as Vowel, Thyroid, and QWS. As a result, MDNN's classification performance is more constant than that of other methods. Nevertheless, MDNN appears to have a minor disadvantage on the large datasets, such as Balance scale, Vehicle, CMC, and Robot navigation, which seem to be constrained by the distribution of the network structure. In the comparison between MDNN and other classifiers, the superiority and effectiveness of the multiple dendritic neuron structure are verified.

**Figure 6:**
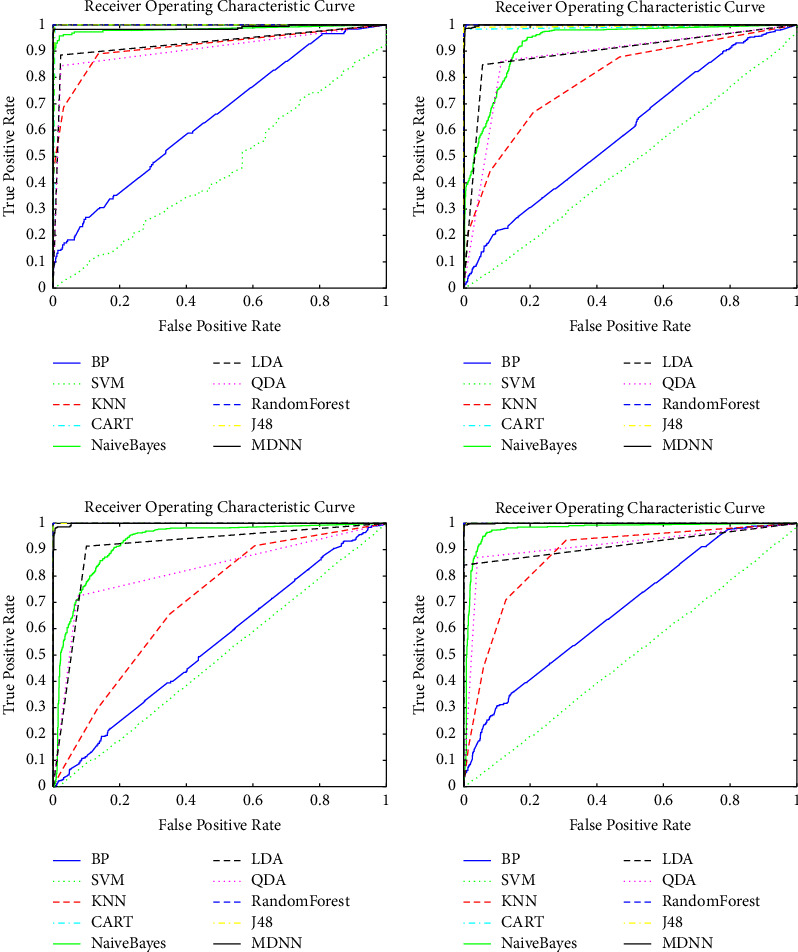
(a) The comparison of receiver operating characteristic curves for the platinum class of the QWS dataset. (b) The comparison of receiver operating characteristic curves for the gold class of the QWS dataset. (c) The comparison of receiver operating characteristic curves for the silver class of the QWS dataset. (d) The comparison of receiver operating characteristic curves for the bronze class of the QWS dataset.

The receiver operating characteristic (ROC) curves of ten multiclassification methods show the correct classification coverage of each class of the QWS dataset in Figure 6. It can be found that MDNN not only has a consistent performance on each class of QWS but also outperforms the other classifiers, thus indicating the effectiveness and stability of MDNN for the QWS classification and unbalanced multiclassification applications. Besides, experiments also demonstrate the efficiency and superiority of MDNN in terms of classification performance and stability.

### 4.4. Morphology and Logical Circle Realization

**Figure 7:**
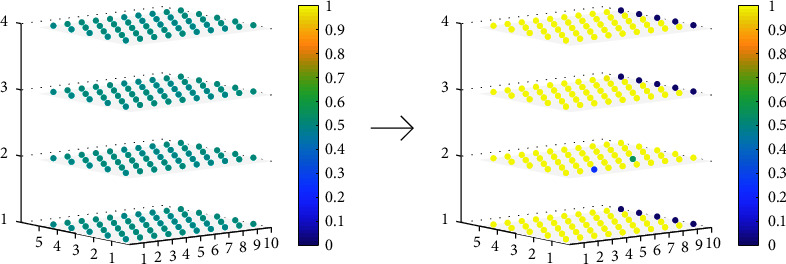
Dendritic changes in the structure of randomly selected samples before and after network training were applied to the QWS dataset. *Z*-axis represents the neuron, and *X*-axis and *Y*-axis are attributes and hidden layers, respectively.

For the display of a data sample, the shuffle operation set in the pretraining period is at disposal. According to the initialization of *w*_*j*,*i*,*m*_ and *q*_*j*,*i*,*m*_ within Section 2, the synapses were calculated around 0.5 in the previous state. Through the training stage, the weights *w*_*j*,*i*,*m*_ and *q*_*j*,*i*,*m*_ were gradually stabilized. As shown in Figure 7, synaptic changes, thus, yield to accomplish the pruning of the redundant network structure.

It can be easily observed that neuron 1, neuron 2, and neuron 4 are fully inhibited in accordance with the rule of Equation. (2) and Equation. (3). Consequently, the structure of neuron 1, neuron 3, and neuron 4 is ruled out. To specify the states of dendrites, a total of four scenarios are listed as follows:

(28) $$\begin{array}{c} \text{dendritic state}=\left\{\begin{array}{cc} \text{constant}-1\;\text{connection}, & \text{when }q_{j,i,m}<w_{j,i,m}<0\text{ or }q_{j,i,m}<0<w_{j,i,m,}\\ \text{constant}-0\;\text{connection}, & \text{when }0<w_{j,i,m}<q_{j,i,m}\text{ or }w_{j,i,m}<0<q_{j,i,m,}\\ \text{excitatory }\text{connection}, & \text{when }0<q_{j,i,m}<w_{j,i,m,}\\ \text{inhibitory }\text{connection}. & \text{when }w_{j,i,m}<q_{j,i,m}<0\text{.} \end{array}\right. \end{array}$$

**Figure 8:**
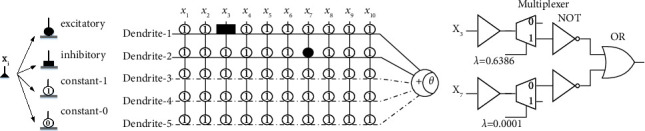
(a) The dendritic states of MDNN are fixated on the QWS dataset. Circle 0 and Circle 1 stand for constant-0 and constant-1 connection, respectively. In addition, black-filled square and circle stand for inhibitory and excitatory states, respectively. (b) Logical circle realization of MDNN on the QWS dataset, where *λ* = *q*_*j*,*i*,*m*_/*w*_*j*,*i*,*m*_.

For the remainder neuron, the residual dendrite morphology is formed in Figure 8(a), which places the line of dashes indicating pruning. As mentioned previously in Section 2, logic-based inherent relations have existed within dendritic structures. Finally, since constant-1 holds no substantive impact on the attributes, the connections among dendrites are equivalent to logic OR. Thus, the hardware realization is transformed as illustrated in Figure 8(b), where the multiplexer is a 1 : 2 numerical compactor. In addition to showing the extendibility of MDNN, the overhead also indicates that MDNN can avoid overfitting by increasing the dendritic matrix sparsity.

## 5. Conclusion and Future Directions

This article puts forward a novel extended network of dendritic neurons, namely, the multiple dendritic neural network (MDNN). The architecture of MDNN is completely different from the previous DL models which are based on MP neuron models. By deriving its new learning algorithms, MDNN is for the first time able to resolve the multiclassification problems in comparison with previous single dendritic neuron models. Besides, we propose an approach to improve the interpretability of artificial neural networks with the theoretical support of neuroscience. Experiments are mainly carried out on a QoS-related application. In the comparison between MDNN and other classifiers, the superior performance of the proposed model is shown, and MDNN is also highly advantageous to small-scale unbalanced data. In view of this, the performance and efficiency of the proposed neural network are limited by scale. In the follow-up work, the deficiency of this experiment will be made up to improve the generalization ability [45, 46] and study the capabilities and limitations. Meanwhile, the exploration of applicable domains for MDNN will be conducted in the following aspects: 1) expanding research on more computer-related data mining to solve practical engineering problems, such as quality of service of mobile networks [47] and security bug report [48, 49]; 2) practicing in other forms of data structures, e.g., semantic [50]; 3) focusing on the unbalanced data [51] and simplifying the network structure adequately [52] with the practice.

## Figures and Tables

**Table 1:** Hyperparameters in the experiments.

ID	Parameters	Description	Default
1	activation	Activation function	Sigmoid
2	*k*	Parameter for Leaky ReLU	0.01
3	time	Operation times	30
4	*η*	Learning rate	0.01
5	*M*	Number of nodes for the hidden layer	5
6	epoch	Training epoch	100
7	batchsize	Mini-batch size for training	100
8	precision	End the training if reaching the precision	0.1
9	Ada m	Active or inactive the optimization	1
10	*α*	Parameter for Adam	0.9
11	*β*	Parameter for Adam	0.999
12	epsilon	Parameter for Adam	1e-8

**Table 2:** Orthogonal experimental design of hyperparameters with *L*_9_(3^4^) for QWS.

No.	batchsize	*M*	precision	*η*
1	120	4	0.01	0.005
2	120	5	0.02	0.008
3	120	6	0.05	0.01
4	240	4	0.02	0.01
5	240	5	0.05	0.005
6	240	6	0.01	0.008
7	364	4	0.05	0.008
8	364	5	0.01	0.01
9	364	6	0.02	0.005

**Table 3:** Optimal parameter settings for QWS.

ID	Parameters	Optimal	Adaptive/Not adaptive
1	batchsize	364	Not adaptive
2	precision	0.01	Not adaptive
3	*M*	5	Not adaptive
4	*η*	0.01	Not adaptive
5	epoch	100	Adaptive

**Table 4:** Average and optimum values of classification results of MDNN for QWS.

		Platinum	Gold	Silver	Bronze	Mean values
Precision	(Average)	99.44	98.48	98.92	99.55	99.10
	(Optimum)	100.00	100.00	100.00	100.00	100.00

Recall	(Average)	96.52	99.72	99.46	98.81	98.63
	(Optimum)	100.00	100.00	100.00	100.00	100.00

F1	(Average)	97.73	99.05	99.16	99.15	98.77
	(Optimum)	100.00	100.00	100.00	100.00	100.00

Accuracy	(Average)	99.58	99.52	99.45	99.52	99.52
	(Optimum)	100.00	100.00	100.00	100.00	100.00

AUC	(Average)	99.11	99.71	99.92	99.97	99.68
	(Optimum)	100.00	100.00	99.85	100.00	99.96

**Table 5:** Description of datasets and parameter settings of MDNN.

Datasets	Instances	Number of features	Number of classes	Size of classes	*M*	*η*
Iris	150	4	3	50, 50, 50	4	0.01
Wine	178	13	3	59, 71, 48	5	0.01
Vehicle	846	18	4	199, 217, 218, 212	10	0.02
Balance scale	625	4	3	49, 288, 288	12	0.01
CMC	1473	9	3	629, 333, 511	20	0.03
Seed	210	7	3	70, 70, 70	5	0.01
Vowel	871	3	6	72, 89, 172, 151, 207, 180	6	0.01
Thyroid	215	5	3	150, 35, 30	4	0.01
Robot navigation	5456	24	4	82, 620, 972, 205, 329	30	0.02
QWS	364	10	4	41, 100, 120, 103	5	0.01

**Table 6:** Comparison between MDNN and nine classifiers.

	Precision	Recall	F1	Accuracy	AUC	Precision	Recall	F1	Accuracy	AUC	Precision	Recall	F1	Accuracy	AUC
	*BP*					*SVM*					*KNN*				
Iris	96.30	96.13	95.91	97.39	99.68	96.43	96.11	96.06	97.39	99.76	96.46	96.52	96.29	97.68	98.78
Wine	27.74	43.30	31.65	65.60	62.29	43.62	40.25	31.31	64.28	72.29	71.02	71.02	69.10	80.74	87.78
Vehicle	12.26	28.46	14.91	63.57	54.03	12.11	24.65	10.29	61.77	38.12	62.86	64.22	62.19	82.18	86.18
Balance scale	67.50	67.84	66.37	93.33	90.65	65.70	66.60	65.36	91.35	94.59	59.70	56.52	57.24	83.43	65.29
CMC	37.23	42.85	38.26	65.69	64.15	54.45	51.64	51.69	70.37	72.06	47.22	46.01	45.06	66.06	65.75
Seed	90.79	90.43	89.89	93.54	97.56	90.39	90.24	89.98	93.47	98.82	89.05	88.72	88.02	92.43	96.36
Vowel	3.82	16.67	6.21	74.31	41.99	83.78	53.21	57.54	86.21	80.74	83.62	83.27	82.88	94.82	95.55
Thyroid	73.84	71.61	70.86	90.56	87.97	68.72	74.57	67.03	89.79	96.47	94.36	84.25	87.69	95.28	96.81
Robot navigation	73.76	63.39	65.32	88.18	91.38	90.18	87.52	88.73	94.76	98.17	85.03	84.86	84.73	92.68	95.79
QWS	17.64	30.71	19.42	68.94	51.34	85.03	84.86	84.73	92.68	32.69	62.16	60.49	59.39	78.61	81.92

	*CART*					*Naive Bayes*					*LDA*				
Iris	96.26	96.16	96.02	97.20	97.82	95.97	95.87	95.52	97.49	99.52	98.07	98.13	97.94	98.65	99.07
Wine	91.54	92.54	91.36	94.57	95.15	96.35	96.95	96.34	97.70	99.89	97.89	98.20	97.89	98.68	98.55
Vehicle	68.91	69.18	68.73	84.46	84.26	51.67	46.11	41.68	72.66	77.41	77.63	78.53	77.71	89.23	87.98
Balance scale	55.75	57.27	56.25	85.72	76.35	60.99	65.62	62.88	93.22	89.66	73.68	80.32	67.58	82.65	82.20
CMC	49.79	49.23	49.15	67.63	67.37	49.45	49.88	47.56	65.12	67.56	49.88	50.52	49.06	66.44	63.64
Seed	88.81	88.56	87.99	92.29	92.98	91.42	91.41	91.14	94.24	98.67	96.77	96.76	96.65	97.92	97.59
Vowel	96.77	96.76	96.65	97.92	97.59	77.72	77.50	76.89	93.22	96.75	75.36	76.85	75.06	92.62	84.00
Thyroid	87.46	88.22	86.31	94.58	93.00	97.34	95.77	96.15	98.26	99.65	95.81	88.15	90.53	95.69	93.08
Robot navigation	**99.17**	99.22	**99.19**	**99.67**	99.59	54.97	66.44	54.20	76.36	83.66	56.80	66.73	58.53	80.82	75.68
QWS	98.18	98.71	98.40	**99.73**	98.64	81.61	84.12	81.71	90.64	95.78	86.35	86.35	85.77	93.42	89.55

	*QDA*					*J48*					*Random Forest*				
Iris	97.60	97.61	97.44	98.36	98.82	100.00	97.78	98.83	94.07	98.89	**100.00**	**100.00**	**100.00**	94.81	**100.00**
Wine	98.95	98.60	98.67	99.18	99.18	92.54	93.19	92.63	91.57	94.68	97.15	98.12	97.56	96.26	99.82
Vehicle	85.20	85.49	85.02	92.57	92.19	86.67	89.89	88.15	71.60	94.06	**87.47**	**95.21**	**91.10**	74.88	**99.09**
Balance scale	**83.57**	**91.04**	**85.62**	94.47	91.71	3.82	2.71	3.03	77.92	53.65	2.75	3.40	3.02	80.33	53.38
CMC	**62.28**	60.57	60.86	**74.96**	**78.12**	61.36	61.25	61.20	51.94	69.31	59.32	**64.59**	**61.77**	50.67	71.65
Seed	94.94	94.40	94.48	96.39	95.22	86.98	86.83	86.59	91.06	89.59	87.91	90.32	88.92	92.49	96.11
Vowel	75.47	77.50	75.50	92.80	84.42	63.17	57.64	59.30	83.52	86.94	64.81	64.99	64.23	85.11	89.00
Thyroid	94.20	95.29	94.17	97.29	97.17	94.39	95.11	94.67	92.51	91.31	95.43	98.37	96.84	95.50	98.61
Robot navigation	64.42	75.59	66.73	83.44	82.74	98.90	99.37	99.13	99.40	99.67	98.61	**99.42**	99.01	99.41	**99.98**
QWS	83.65	85.18	83.70	91.52	89.31	97.99	**100.00**	**98.95**	99.76	99.86	97.04	96.75	96.78	98.99	**99.93**

	*MDNN*														
Iris	**100.00**	**100.00**	**100.00**	**100.00**	**100.00**										
Wine	**100.00**	**100.00**	**100.00**	**100.00**	**100.00**										
Vehicle	85.34	85.48	85.33	**93.31**	96.12										
Balance scale	80.50	77.76	78.92	**95.74**	**93.26**										
CMC	**62.28**	60.57	60.86	**74.96**	**78.12**										
Seed	**100.00**	**100.00**	**100.00**	**100.00**	**100.00**										
Vowel	**91.29**	**84.26**	**86.47**	**95.67**	**98.03**										
Thyroid	**100.00**	**100.00**	**100.00**	**100.00**	**100.00**										
Robot navigation	94.89	92.11	93.41	97.19	99.30										
QWS	**99.10**	98.63	98.77	99.52	99.68										

## Data Availability

The classification dataset could be downloaded freely at https://archive.ics.uci.edu/ml/index.php.
